# Spontaneous variability of pre-dialysis concentrations of uremic toxins over time in stable hemodialysis patients

**DOI:** 10.1371/journal.pone.0186010

**Published:** 2017-10-10

**Authors:** Sunny Eloot, Wim Van Biesen, Sanne Roels, Willem Delrue, Eva Schepers, Annemieke Dhondt, Raymond Vanholder, Griet Glorieux

**Affiliations:** 1 Department of Nephrology, Ghent University Hospital, Gent, Belgium; 2 Department of Data Analysis, Ghent University, Gent, Belgium; University of Glasgow, UNITED KINGDOM

## Abstract

**Background and aim:**

Numerous outcome studies and interventional trials in hemodialysis (HD) patients are based on uremic toxin concentrations determined at one single or a limited number of time points. The reliability of these studies however entirely depends on how representative these cross-sectional concentrations are. We therefore investigated the variability of predialysis concentrations of uremic toxins over time.

**Methods:**

Prospectively collected predialysis serum samples of the midweek session of week 0, 1, 2, 3, 4, 8, 12, and 16 were analyzed for a panel of uremic toxins in stable chronic HD patients (N = 18) while maintaining dialyzer type and dialysis mode during the study period.

**Results:**

Concentrations of the analyzed uremic toxins varied substantially between individuals, but also within stable HD patients (intra-patient variability). For urea, creatinine, beta-2-microglobulin, and some protein-bound uremic toxins, Intra-class Correlation Coefficient (ICC) was higher than 0.7. However, for phosphorus, uric acid, symmetric and asymmetric dimethylarginine, and the protein-bound toxins hippuric acid and indoxyl sulfate, ICC values were below 0.7, implying a concentration variability within the individual patient even exceeding 65% of the observed inter-patient variability.

**Conclusion:**

Intra-patient variability may affect the interpretation of the association between a single concentration of certain uremic toxins and outcomes. When performing future outcome and interventional studies with uremic toxins other than described here, one should quantify their intra-patient variability and take into account that for solutes with a large intra-patient variability associations could be missed.

## Introduction

Chronic kidney disease is characterized by the retention of numerous solutes, which are at the origin of a deterioration of multiple biochemical and physiological functions [1, 2]. In order to decrease these concentrations on the long term, and with it their biological toxicity, interventions interacting on the generation and/or removal of these uremic toxins have repeatedly been explored. In hemodialysis patients, numerous cross-sectional studies evaluated the association of a single concentration of a specific uremic toxin(s) with patient outcomes [3–9]. Also, various interventional studies investigated the impact of a change in dialysis prescription on different toxin concentrations based on a limited number of measurements [10–14]. However, a substantial spontaneous background fluctuation of concentrations of uremic toxins within one patient over time might have an impact on the interpretation of this type of studies, whereby the effect can be both over- or underestimated purely based on chance.

Up till now, it has not been investigated in a systematic way whether predialysis concentrations of uremic toxins remain constant in stable hemodialysis patients over a given time period. The present study aimed at evaluating: 1) the intra-patient variability over time in comparison with the inter-patient variability of concentrations of a panel of uremic toxins; and 2) the impact on concentration variability of patient-related and/or dialysis-related characteristics. We therefore quantified the variability of predialysis uremic toxin concentrations in hemodialysis patients over a period of 16 weeks.

## Methods

### Patients and dialyses

The study included eighteen stable chronic hemodialysis patients (3 women, 11 with diabetes mellitus) of 72.5±10.1 years old, 55.7±30.1 months on dialysis, and with a residual renal function (as calculated from the arithmetic mean of the creatinine and urea clearance [15]) of 3.5±3.0mL/min. Twelve patients had a well-functioning arteriovenous fistula as vascular access, five patients a Palindrome 14.5F double lumen central venous catheter (Covidien, USA), and one patient had a synthetic graft combined with a single lumen Tesio 12F central venous catheter (Medcomp, USA). Monthly monitoring of the access flow showed no access recirculation in the arteriovenous fistulae. Exclusion criteria were pregnancy, unstable condition, vascular access problems, and age below 18. The study was designed according the Declaration of Helsinki, approved by the local Ethics Committee, and written informed consent was obtained from all participants (Ghent University Hospital: EC2012/603; B670201214999).

The study period lasted for 16 weeks with a test session on midweek 0, 1, 2, 3, 4, 8, 12, and 16. During the entire study period (i.e. for all patients lasting from September to January), the dialysis mode, dialyzer blood and dialysate flows and hemodialyzer type were maintained stable in each patient, i.e. two-needle/lumen post dilution hemodiafiltration (except in one patient who received hemodialysis) with high flux dialyzers: FX800 (n = 12) (Fresenius Medical Care, Germany), Phylter HF 17G (n = 2) (Bellco, Italy), Sureflux 170 (n = 1) (Nipro Europe, Belgium), Xenium 210 (n = 1), Polyflux 170H (n = 1) and Evodial 1.3 (n = 1) (all three from Baxter, USA). During the test sessions, blood and dialysate flows were 311±21 and 530±39mL/min, respectively, while ultrafiltration (i.e. referring to the weight loss of the patient during dialysis) was set according to the need of the patient and could vary over the study period. Kt/V_urea_ was monthly assessed using the single pool Daugirdas formula [16].

### Sampling and analysis

At the midweek test sessions, blood samples (Venosafe, gel+clot act.; Terumo Europe N.V., Leuven, Belgium) were collected predialysis from the vascular access and were centrifuged after 20-30min to allow clotting (10min at 1250g, 4°C), after which the serum was aliquoted and stored at -80°C until batch analysis within 3 months after the last sample collection. We previously showed that the detected concentrations of uremic toxins is not influenced by a single freeze/thaw cycle [17].

Urea (molecular weight MW: 60Da), creatinine (Crea, 113Da), and phosphorus (P, 31Da) were measured by standard laboratory methods in the routine laboratory of the Ghent University Hospital, assuring measurement reproducibility. Urea was determined by Cobas c701(a+b) urease UV kinetic measurement (Roche, Switzerland), creatinine by a Cobas c systems 2012–02 V10 Enzymatic modified Jaffé method (Roche, Switzerland), and phosphorus by Cobas c701(a+b) molybdaat UV (Roche, Switzerland).

Other solutes were determined by reversed-phase high performance liquid chromatography (RP-HPLC), as described earlier [13, 18]. These included uric acid (UA, 168Da), and the protein-bound solutes p-cresylglucuronide (PCG, 284Da), hippuric acid (HA, 179Da), indole acetic acid (IAA, 175Da), indoxyl sulfate (IS, 213Da), p-cresylsulfate (PCS, 187Da), and 3-carboxy-4-methyl-5-propyl-2-furanpropionic acid (CMPF, 240Da). To determine the total concentration, serum samples were first deproteinized by heat denaturation (95°C, 30 min). After heating, the samples were placed on ice for 10 minutes. Subsequently, the samples were centrifuged (7379 x g, 10 min) and filtered (3615 x g, 20 min, room temperature) through Amicon Ultra 0.5 mL Filters (molecular weight cut-off 30 kDa, Merck KGaA, Darmstadt, Germany). With HPLC, HA and CMPF were analyzed by UV detection at 254nm, and UA at 300nm, whereas *p*CG and *p*CS (λexc = 265nm, λem = 290nm) and IAA and IS (λexc = 280nm, λem = 340nm) were determined by fluorescence detection [13, 18]. To obtain free fractions, untreated serum samples were filtered through a Centrifree^®^ filter device (Millipore Billerica, MA, USA) prior to heating.

Recent validation of our HPLC analysis method showed intra-day precision (% relative standard deviation; RSD) for UA, pCG, HA, IAA, IS, PCS and CMPF of 1.92, 2.39, 1.99, 2.56, 2.58, 2.52, and 8.59%, respectively, and an inter-day precision of 3.65, 6.38, 3.24, 6.34, 4.84, 5.95, and 11,57%, respectively, in pooled samples from CKD patients.

Enzyme-linked immunosorbent assay (ELISA) kits manufactured by DLD Diagnostika GmbH (Hamburg, Germany) were used to quantify symmetric dimethylarginine (SDMA) and asymmetric dimethylarginine (ADMA) (both MW 202Da). For beta-2-microglobulin (β_2_M) (11800Da), ELISA kits of Orgentec Diagnostika GmbH (Mainz, Germany) were used. Manufacturer’s data report intra-assay coefficient of variation between 3.8–4.9% for β_2_M, 4.7–6.1% for SDMA, 5.and 7–6.8% for ADMA.

### Statistical analysis

For all concentrations and based on a linear mixed model that accounted for differences in gender, age, BMI, dialysis vintage, Kt/V, UF (ultrafiltration), RRF (residual renal function), and DM (diabetes mellitus) via the inclusion of covariates, the Intra-class Correlation Coefficient (ICC) was calculated as ICC = (inter-patient variability)^2^ / [(inter-patient variability)^2^ + (intra-patient variability)^2^] = inter-patient variance / (inter-patient variance + intra-patient variance). A threshold of ICC≥0.7 was assumed [19], corresponding to an intra-patient variability of maximum 65% of inter-patient variability (as derived from the above mentioned equation).

The covariates (gender, age, BMI, dialysis vintage, Kt/V, UF, RRF, and DM) were checked for independency by a correlation analysis.

In the calculation of the ICC, administration of antibiotics was considered in cases when blood sampling was performed at least 2 days after the first antibiotics intake, and in the statistical analysis, the effect of the antibiotics was assumed to last until the end of the study.

Time was entered in the mixed model via dummy variables to check for any type of trend over time. Via a model comparison with and without time the effect of time was assessed.

Relations between uremic toxin concentrations with patient and dialysis characteristics (i.e. gender, age, BMI, dialysis vintage, Kt/V, UF, RRF, and DM) were checked via the mixed models.

## Results

At the start of the study period, patients had a BMI of 25.2±2.9 and single pool Kt/V of 1.5±0.3.

**Fig 1:**
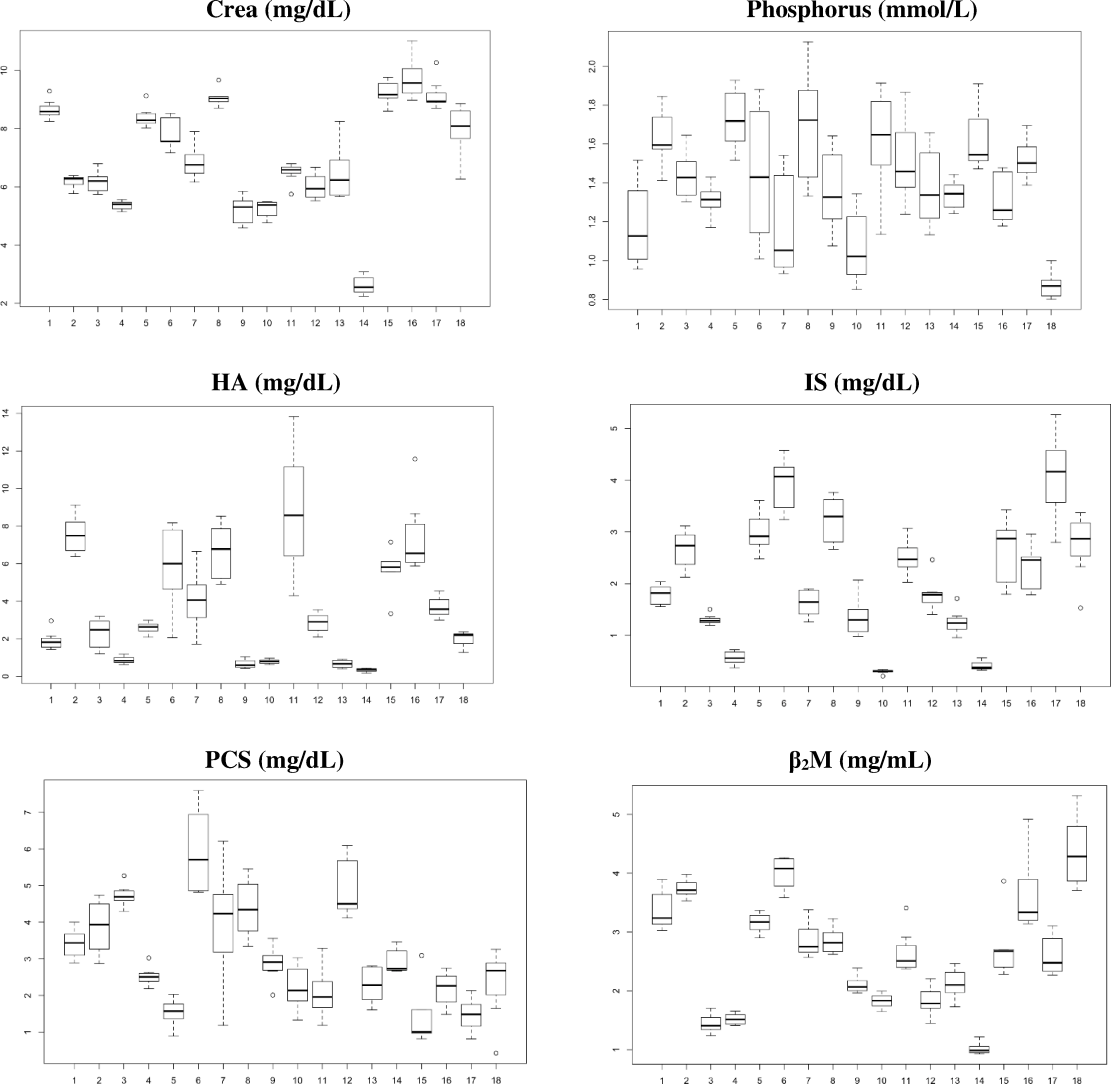
Inter-patient variability. Box plots of predialysis serum concentrations on the respective original scales (y-axis) over the 18 patients (x-axis): Crea (creatinine), phosphorus, total HA (hippuric acid), total IS (indoxyl sulfate), total PCS (p-cresylsulfate), and β_2_M (beta-2-microglobulin).

**Fig 2:**
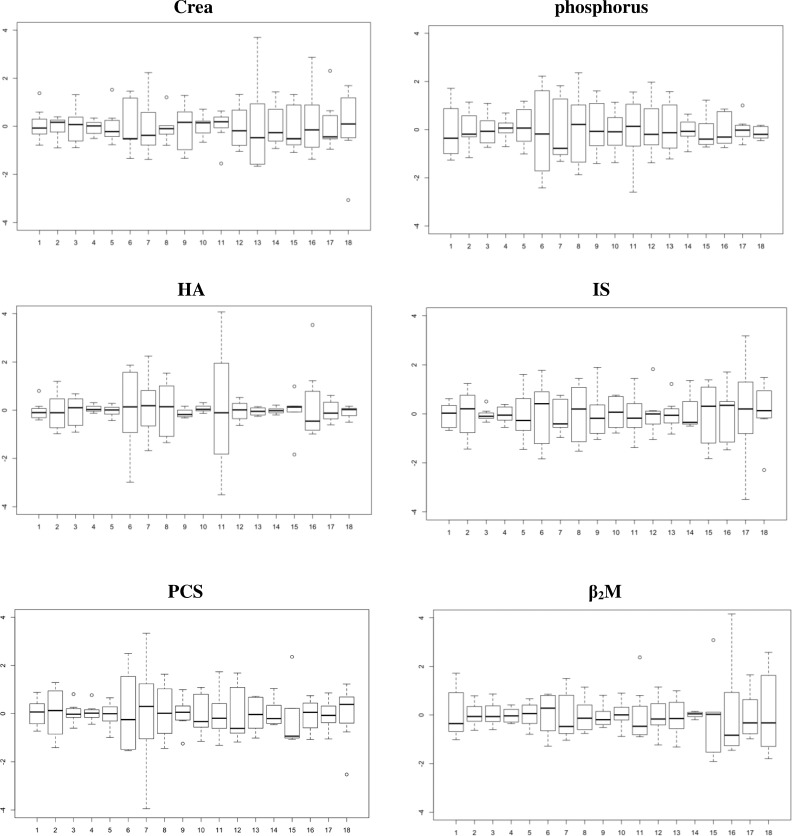
Intra-patient variability. Box plots of predialysis serum concentrations, re-scaled in such a way that within patients (x-axis) the mean is 0 and that over the patients the SD = 1 (y-axis): for Crea (creatinine), phosphorus, total HA (hippuric acid), total IS (indoxyl sulfate), total PCS (p-cresylsulfate), and β_2_M (beta-2-microglobulin).

Concentrations of different studied uremic toxins in the 18 patients highlight the substantial inter-patient variability (Fig 1). In Fig 2, concentrations are centered per patient (mean was set at zero within subjects and SD = 1; y-axis). By aligning the different patients, the intra-patient variability is illustrated. The variability around the median (bold line) is patient dependent and not in the same range for the different studied uremic toxins.

Table 1 shows the Intra-class Correlation Coefficients (ICCs), the overall average toxin concentration, and the inter- and intra-patient variances. ICC was corrected for gender, age, BMI, dialysis vintage, Kt/V, UF (ultrafiltration), RRF (residual renal function), and diabetes status, and was accounted for antibiotic administrations. For urea, creatinine, β_2_M, and some protein-bound uremic toxins (total concentrations of PCG, IAA, PCS, and CMPF; and all free fractions except for HA), ICC was higher than 0.7 indicating that the intra-patient variability was less than 65% of the inter-patient variability. For phosphorus, UA, SDMA, ADMA, and the protein-bound toxins HA (total) and IS (total and free concentrations), however, ICC values range between 0.5 and 0.7 implying a substantial concentration variability within the patient.

**Table 1:** Intra-class correlation coefficients (ICC) corrected for different covariates, with overall toxin average concentration, and inter- and intra-patient variances.

toxin	ICC	average	inter-patient variance	intra-patient variance
urea	0.74	99.6	606	207
creatinine	0.75	6.98	0.685	0.232
phosphorus	**0.58**	1.39	0.048	0.035
SDMA	**0.69**	0.89	0.035	0.015
ADMA	**0.69**	2.63	0.290	0.128
UA	**0.64**	7.09	0.911	0.509
PCG	0.86	0.72	0.455	0.075
HA	**0.50**	3.50	1.616	1.586
IAA	0.81	0.21	0.033	0.008
IS	**0.63**	2.05	0.237	0.137
PCS	0.79	3.06	1.821	0.475
CMPF	0.94	0.33	0.109	0.007
Free PCG	0.85	0.65	0.378	0.068
Free HA	**0.55**	2.15	1.095	0.881
Free IAA	0.84	0.07	0.006	0.001
Free IS	0.83	0.15	0.011	0.002
Free PCS	0.85	0.19	0.030	0.005
β_2_M	0.76	2.64	0.317	0.101

ICC < 0.70 is indicated in bold; all average concentrations in mg/dL except phosphorus (mmol/L), SDMA and ADMA (both μmol/L) and β_2_M (mg/mL).

SDMA: symmetric dimethylarginin; ADMA: asymmetric dimethylarginin; UA: uric acid; PCG: p-cresylglucuronide; HA: hippuric acid; IAA: indole acetic acid; IS: indoxyl sulfate; PCS: p-cresylsulfate; CMPF: 3-carboxy-4-methyl-5-propyl-2furanpropionic acid; β_2_M: beta-2-microglobulin

**Fig 3:**
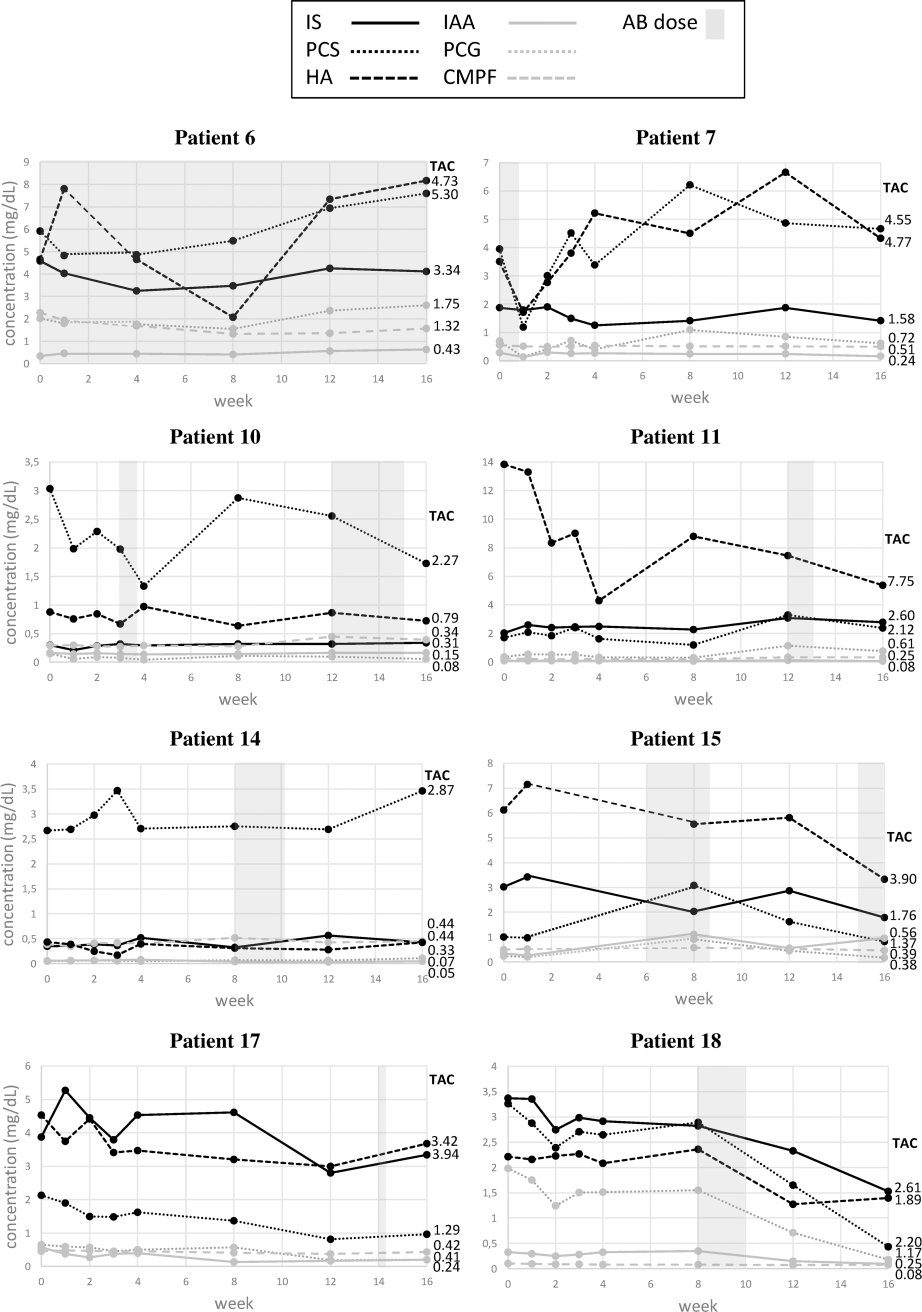
Concentration profiles in patients on antibiotics for the protein-bound toxins PCG (p-cresylglucuronide), HA (hippuric acid), IAA (indole acetic acid), IS (indoxyl sulfate), PCS (p-cresylsulfate), and CMPF (3-carboxy-4-methyl-5-propyl-2-furanpropionic acid). The antibiotics interval is indicated in grey shadow and the time averaged concentration (TAC) per uremic toxin and per patient is added to the right side of each graph. Concentrations in mg/dL, except phosphorus (mmol/L), SDMA and ADMA (both μmol/L) and β_2_M (mg/mL).

During the study period, 8 patients received antibiotics (Table 2 and Fig 3). No significant differences were found between the ICC values with or without accounting for antibiotics therapy, although in 4 out of 8 patients, effects of antibiotic therapy on concentrations of PCS and/or IS can be observed (Fig 3).

**Table 2:** Antibiotics therapy.

patient	Start of administration (study week)	Duration (days)	antibiotics
6	Before study start	continuous	Temocillin 2g (3x/week postdialysis)
7	0	5	amoxicillin 500mg + clavulanic acid 125mg (3/day)
10	3	6	amoxicillin 500mg + clavulanic acid 125mg (3/day)
10	12	21	amoxicillin 500mg (3/day)
11	12	5	amoxicillin 500mg + clavulanic acid 125mg (3/day)
14	8	16	amoxicillin 500mg + clavulanic acid 125mg (3/day)
15	6	19	vancomycin (target > 25mg/L)
15	15	10	amoxicillin 500mg (3/day)
17	14	2	amoxicillin 2g + clavulanic acid 200mg (2/day)
18	8	14	amoxicillin 500mg + clavulanic acid 125mg (3/day)

Since concentrations for patient 6 were more than 2 SDs larger than the overall mean concentration for free IAA, free PCS, total CMPF and free IS, patient 6 could be assumed being an outlier. However, differences in ICC were less than 10% with or without considering patient 6, except for the free fractions of IS and PCS (decrease of ICC by 19 and 27%, respectively).

No trend over time was noticed for the different uremic toxin concentrations, except for β_2_-microglobulin with slightly increasing concentrations (0.02mg/mL) over the 16 weeks (P<0.001).

The concentrations of none of the uraemic toxins did show any relation with patient and dialysis characteristics after adjusting for multiple testing.

## Discussion

The present study investigated how intra-patient variability over time is related to the inter-patient variability of concentrations of different uremic toxins in stable hemodialysis patients, and whether these variabilities are influenced by patient and/or dialysis related characteristics. Eighteen stable hemodialysis patients were followed during 16 weeks and predialysis midweek blood samples were analyzed for different levels of uremic toxins.

Our main findings are that 1) intra-patient variability was most pronounced for phosphorus, SDMA, ADMA, UA, total and free HA and total IS, 2) antibiotics did not play a significant role in the overall concentration variability but seemed to have an impact in some individuals, and 3) no consequent relation was found between toxin concentrations and different patient and dialysis related characteristics.

With variations up to 4 SDs, an important inter-patient variability for all studied solutes was demonstrated. Based on a previous multifactor analysis in 71 patients, the inter-patient variabilities found in the present study could for different toxins be attributed to differences in PNA between the individual patients [15]. In addition, since several of these uremic toxins (PCS and IS) are from colonic origin, variation and changes in the composition and metabolic function of the gut microbiota might play a role affecting generation [20]. And it is known that gut microbiota composition is influenced by many factors characteristic for hemodialysis patients such as older age, medication (antibiotics), nutrition, and exercise [21]. However the link between toxin concentration and specific microbiota in CKD needs further investigation.

It is however more difficult to explain the observed intra-patient variability. Since most patient and dialysis related characteristics were constant in our study period of 16 weeks, age, gender, diabetes mellitus, BMI, dialysis vintage, ultrafiltration nor Kt/V cannot explain the observed variability. As we did not control for it, and as it is considered as an important parameter impacting on uremic toxin concentrations, changes in nutritional intake might account for some of the variability. A randomized controlled trial in 29 healthy controls revealed that a high protein intake for 2 weeks resulted in an increase of plasma levels for IS, while no difference was found for the other tryptophan metabolite IAA, and the phenolic compounds PCS and PCG [22]. Furthermore, in a cross-over randomized trial in 31 patients (SYNERGY), synbiotic therapy (i.e. co-administration of pre- and probiotics) over 6 weeks resulted in a significant and potentially clinically important reduction in serum concentration of PCS [23], whereas for IS, this decrease was only found in patients not on antibiotic treatment. This is in analogy with previous findings of a decrease of serum concentrations of p-cresol, the precursor of PCS, with synbiotic therapy [24, 25]. Our results are in line with these studies, as they demonstrate deviating results for the two protein-bound solutes IS and PCS, and thus support that nutrition, or factors affecting metabolism of ingested nutrients (e.g. intestinal microbiota impacting amino acid processing) might play a substantial role in intra-patient variability of serum concentrations of uremic toxins.

Some uremic toxins showing an important intra-patient variability in the present study, i.e. UA, ADMA, SDMA, and IS, have repeatedly been associated with vascular damage and mortality [6, 8, 26–28]. Since these outcome studies are based on cross-sectional blood sampling at isolated time points, it might strengthen their reliability if, despite large intra-patient variability, associations can be found. However, studies considering associations between concentrations of highly variable toxins and outcome risk to miss significance due to the large intra-patient variability.

On the other hand, previous longitudinal studies [11–14], investigating the impact of interventions on toxin concentrations in dialysis patients, are gaining importance since the here called ‘stable’ solutes showed significant concentration changes due to the intervention, while changes were unexplainably absent for solutes with an important intra-patient variability, like HA and IS. Furthermore, the discrepancy in the behavior of solutes that were expected to behave alike (like expected for PCS and IS), can now very likely be explained by differences in intra-patient variability, as reported in this analysis.

The observed and here presented inter- and intra-patient variances allow the calculation of sample sizes for future studies on these uremic toxins. Furthermore, these data can also be of help to setup appropriate study designs, accounting for the relative importance of both variances.

The present study included the classical blood parameters for HD patients (i.e. urea, creatinine, and phosphorus) as well as representative uremic toxins for each class, i.e. the small water soluble solutes: uric acid, ADMA and SDMA; the middle molecule β_2_M, and the protein-bound solutes: pCG, HA, IAA, IS, pCS, and CMPF. This selection was made with special interest for those toxins with already described associations with outcome in CKD [4–6, 8, 29–32].

All samples collected during the 16 weeks were analyzed in batch. The observed variability in concentrations can thus not substantially be attributed to changes in laboratory procedures, and should thus mainly be originating from patient characteristics.

Another confounding factor is the fact that 8 out of 18 of the otherwise stable HD patients needed antibiotic therapy during the follow-up period, which is to our opinion unavoidable in studies in HD patients. Also in the synbiotic study by Rossi et al, around 30% of patients were started on antibiotics at some time point during the study [23]. Furthermore, our sensitivity analysis including only patients not receiving any antibiotics did not change the conclusion that there was substantial intra-patient variability in concentrations of different uremic toxins. Studies on the impact of antibiotics on uremic toxins are relatively scant. A recent study by Nazzal et al. suggested that indoxyl sulfate and p-cresylsulfate concentrations were decreased at day 2 and day 5 after a single vancomycin administration in hemodialysis patients [33]. This study however contained no untreated control group which might be a drawback in view of the presently shown variability of indoxyl sulfate over time. To our knowledge, it has not been established so far if, and to what extent, antibiotics impact on the variability in toxin concentrations, neither on how fast this effect would take on, or how long it would last.

One can postulate that the variability in free concentrations could be affected from the use of antibiotics competing with the protein-bound uremic toxins in their binding to albumin. We found however no differences in percentages of protein binding of the studied protein-bound toxins after antibiotic administration versus baseline (data not shown).

In line with our findings, Nakazato et al also found high intra-patient variability in serum phosphorus in a study on 384 HD patients who were followed for 11 years [34]. Interestingly, they could link high concentration variability to poor general conditions and non-cardiac mortality of their patients [34], although these authors did not offer a mechanistic explanation for the mechanisms provoking this variability.

In conclusion, this study found, more or less as expected, a substantial inter-patient variability in uremic toxin concentrations among stable HD patients, but also a marked intra-patient variability which might have an impact on evaluations on the association of concentrations and outcomes, as well as on the interpretation of studies assessing the impact of interventions on uremic solute concentrations. With respect to the latter, if changes in concentrations in the individual patient in an A-B or B-A cross-over design are smaller than the intra-patient variability presented in this paper, these random changes after intervention should be considered as ‘clinically irrelevant’. Alternatively, cross-over designs A-B-A and B-A-B could be used to further highlight that indeed the intervention caused the change in concentration, and that this change is wiped out by returning to the alternative strategy. Furthermore, when performing future outcome and interventional studies with uremic toxins other than described here, one should quantify their intra-patient variability and take into account that for solutes with a large intra-patients variability associations could be missed.
